# Predictors of In-Hospital Mortality for Road Traffic Accident-Related Severe Traumatic Brain Injury

**DOI:** 10.3390/jpm11121339

**Published:** 2021-12-09

**Authors:** Chien-Hung Chen, Yu-Wei Hsieh, Jen-Fu Huang, Chih-Po Hsu, Chia-Ying Chung, Chih-Chi Chen

**Affiliations:** 1 Department of Physical Medicine and Rehabilitation, Chang Gung Memorial Hospital, Chang Gung University, Taoyuan 33305, Taiwan; B9002052@cgmh.org.tw (C.-H.C.); chiaying928@yahoo.com (C.-Y.C.); 2 Department of Occupational Therapy and Graduate Institute of Behavioral Sciences, School of Medicine, Chang Gung University, Taoyuan 33302, Taiwan; Ywhsieh@mail.cgu.edu.tw; 3 Healthy Aging Research Center, Chang Gung University, Taoyuan 33302, Taiwan; 4 Department of Traumatology and Emergency Surgery, Chang Gung Memorial Hospital, Chang Gung University, Taoyuan 33305, Taiwan; jenfu0211@yahoo.com.tw (J.-F.H.); m7831@cgmh.org.tw (C.-P.H.)

**Keywords:** traumatic brain injury, children, road traffic accident, mortality, predictors

## Abstract

(1) Background: Road traffic accidents (RTAs) are the leading cause of pediatric traumatic brain injury (TBI) and are associated with high mortality. Few studies have focused on RTA-related pediatric TBI. We conducted this study to analyze the clinical characteristics of RTA-related TBI in children and to identify early predictors of in-hospital mortality in children with severe TBI. (2) Methods: In this 15-year observational cohort study, a total of 618 children with RTA-related TBI were enrolled. We collected the patients’ clinical characteristics at the initial presentations in the emergency department (ED), including gender, age, types of road user, the motor components of the Glasgow Coma Scale (mGCS) score, body temperature, blood pressure, blood glucose level, initial prothrombin time, and the intracranial computed tomography (CT) Rotterdam score, as potential mortality predictors. (3) Results: Compared with children exhibiting mild/moderate RTA-related TBI, those with severe RTA-related TBI were older and had a higher mortality rate (*p* < 0.001). The in-hospital mortality rate for severe RTA-related TBI children was 15.6%. Compared to children who survived, those who died in hospital had a higher incidence of presenting with hypothermia (*p* = 0.011), a lower mGCS score (*p* < 0.001), a longer initial prothrombin time (*p* < 0.013), hyperglycemia (*p* = 0.017), and a higher Rotterdam CT score (*p* < 0.001). Multivariate analyses showed that the mGCS score (adjusted odds ratio (OR): 2.00, 95% CI: 1.28–3.14, *p* = 0.002) and the Rotterdam CT score (adjusted OR: 2.58, 95% CI: 1.31–5.06, *p* = 0.006) were independent predictors of in-hospital mortality. (4) Conclusions: Children with RTA-related severe TBI had a high mortality rate. Patients who initially presented with hypothermia, a lower mGCS score, a prolonged prothrombin time, hyperglycemia, and a higher Rotterdam CT score in brain CT analyses were associated with in-hospital mortality. The mGCS and the Rotterdam CT scores were predictive of in-hospital mortality independently.

## 1. Introduction

Traumatic brain injury (TBI) is among the leading causes of mortality and morbidity in children and, globally, has large impacts on children’s health [1,2,3,4]. As transportation has improved in cities, road traffic accidents (RTAs) have become responsible for a significant proportion of pediatric TBIs. RTAs are the most frequent cause of severe TBI among young children worldwide [5] and are the leading cause of death for children and young adults aged 5–29 years, according to reports by the World Health Organization in 2021 [6].

Prognostic information is valuable for clinicians and family members to guide the therapeutic options and long-term care preparations, especially for those with severe TBI. Clinical characteristics, including young age, specific injury mechanism, a decreased Glasgow Coma Scale (GCS) score, reduced pupil reaction, hypotension, hypothermia, hyperglycemia, coagulation disorders, and neuroimaging discoveries of subdural hemorrhage, subarachnoid hemorrhage, parenchymal hemorrhage, brain edema, and mass effects, are reported predictors of mortality and poor outcomes in pediatric TBI [7,8,9,10,11,12,13,14,15,16]. Most studies have used all injury mechanisms of pediatric TBI, including falls, being struck by/against an object, RTA, assault, and sports, but different injury mechanisms may have different clinical presentations, and predictors of poor outcomes may vary [17,18].

Currently, predictors of mortality in RTA-related TBI are still limited, even though the severity of RTA-related pediatric TBI is often higher than other injury mechanisms [19]. The aim of the present study was to analyze the epidemiological and clinical characteristics in RTA-related TBI children. We also wanted to further evaluate the predictors associated with in-hospital mortality in children with severe TBI. The goal of this study was to demonstrate the different presentations of severe vs. mild or moderate RTA-related TBI, and to improve prognostic counseling and the further care for these children.

## 2. Materials and Methods

### 2.1. Study Design

We conducted a retrospective, observational cohort study in a tertiary trauma center in Taiwan. Children aged 7–18 years old, who were admitted between January 2005 and December 2019 with TBI resulting from RTAs, were enrolled. Patients were eligible if they had been assigned the International Classification of Diseases, Ninth Edition (ICD-9), diagnostic codes 850–854 for intracranial injury and had an injury mechanism of a traffic accident. A physician and a research nurse reviewed all medical records. We excluded patients who died at the accident scene or during transportation. In order to evaluate all the initial presentations following RTAs, those patients who had received first aid in other hospitals and were then transferred more than 24 h after the trauma were excluded. Medical histories were gathered and reviewed from patient charts. In order to focus on the resulting presentation and outcome of RTA-related TBI, we further excluded those patients who also had severe chest and/or abdomen injuries, as identified by an anatomic injury scale (AIS) score > 3 for the chest and abdomen.

Patients were classified with mild to moderate TBI or severe TBI according to the severity of their injury. Injury severity was stratified by the initial GCS score at admission. The GCS, which has a range of 3 to 15, was routinely scored by emergency medical service personnel at the ED. Severe TBI was defined by an initial GCS score ≤ 8. Mild to moderate TBI was defined by an initial GCS score > 8. The Institutional Review Board at Chang Gung Memorial Hospital approved this study: IRB no. 20200050B0.

### 2.2. Variable Definitions

Data were extracted from the medical records of all eligible subjects. The outcome was defined as in-hospital mortality. The parameters selected to evaluate differences between patients with mild to moderate and severe RTA-related TBI included age, gender, type of road user, and mortality. The parameters selected as potential predictors of mortality in patients with severe RTA-related TBI included age, gender, type of road user, and initial clinical presentation to the ED, including the motor component of the GCS (mGCS) score, hypotension, hypothermia, and initial laboratory data, including hyperglycemia, a prolonged prothrombin time, and intracranial CT findings. We selected these reliable parameters based on previous studies that suggested potential risk factors for injury severity and mortality in pediatric TBI [14,20,21,22,23,24,25,26,27,28,29].

We used the mGCS score instead of the full GCS score, as previous studies have shown that the motor component alone is equivalent to the full GCS score for the prediction of survival to hospital discharge [30]. The mGCS was scored from 1 to 6. Hypotension was diagnosed when a patient’s systolic blood pressure (SBP) was below the fifth percentile for their age. Blood pressure less than 70 mmHg + (2* age in years) in children aged 1 to 10 years old, and less than 90 mmHg in children ≥10 years of age, is defined as hypotension, according to the American Heart Association for Cardiopulmonary Resuscitation and Emergency Cardiovascular Care [31]. Children with an initial body temperature below 35 °C were defined as presenting with hypothermia [14]; a prolonged prothrombin time was defined as an international normalized ratio (INR) ≥ 1.2 [20]; and hyperglycemia was defined as a blood glucose level greater than 200 mg/dL on admission to the ED [24]. A physician blinded to the outcome reviewed the CT images obtained in the first twenty-four hours for each patient and assigned a Rotterdam CT score according to the rubric reported by Maas et al. [32].

### 2.3. Statistical Analyses

To analyze the predictors of mortality among children with RTA-related TBI, we compared the differences in clinical characteristics and outcome variables of mild to moderate and severe RTA-related TBI children using descriptive statistics. Comparisons were determined by the Mann–Whitney U test for continuous variables. If any expected cell size was less than 5, comparisons were made using Pearson’s chi-square test, or Fisher’s exact test for categorical variables. Multiple imputation was used to estimate the missing data, which we assumed were missing completely at random. Missing values were filled in with regression-predicted values by generating multiple complete datasets, as this technique permits the analysis of complete datasets [33]. Multivariate logistic regression analyses were conducted to identify potential predictors of in-hospital mortality among children with severe TBI. Univariate analysis was used to identify the candidate predictors with a significance level of *p* < 0.1, and the final multivariate model included only statistically significant predictors with *p*-value < 0.05. Data were entered and analyzed using the STATA version 14.0 software (STATA, Inc., College Station, TX, USA).

## 3. Results

**Figure 1:**
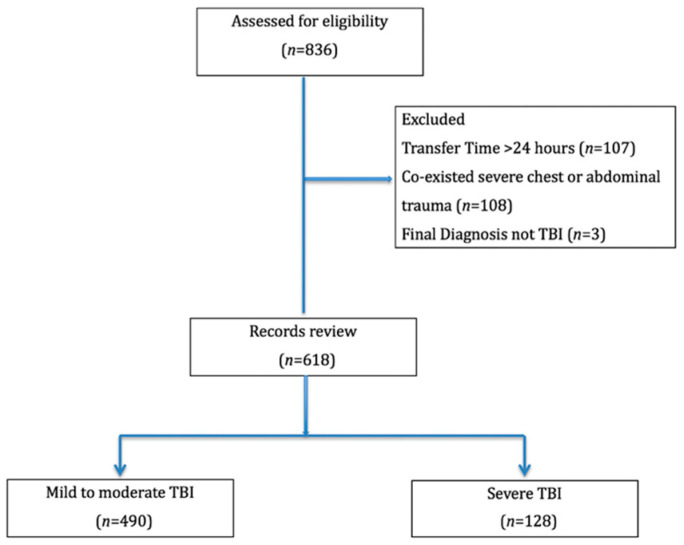
Inclusion and exclusion flow chart.

Of the 836 children initially identified as being admitted for head trauma secondary to head injury, 107 were excluded because their transfer time after the traumatic insult was greater than 24 h, 108 were excluded for co-existing severe chest or abdomen injuries, and 3 had a final diagnosis other than head injury. The resulting study population consisted of 618 patients (Figure 1).

Most of the children were boys (69.09%). Their median age was 16 years old. The type of road user was recorded for 573 patients (92.72%); motorcyclists were the most common (69.28%) (Table 1). A total of 490 patients (79.29%) presented with mild to moderate TBI and 128 (20.71%) with severe TBI. Twenty-three children (3.72%) died in the hospital. Compared to children with mild to moderate TBI, those with severe TBI were older (*p* = 0.004) and had a higher mortality rate (*p* < 0.001). There was no significant difference in gender or mechanism of injury between those with mild to moderate TBI and those with severe TBI (Table 1).

Among children who presented with severe TBI (*n* = 128), 20 (15.63%) died in the hospital (Table 2). Compared to those surviving a severe RTA-related TBI, children with in-hospital mortality presented with a higher incidence of hypothermia (*p* = 0.011), a lower mGCS score (*p* < 0.001), a prolonged initial prothrombin time (*p* < 0.013), hyperglycemia (*p* = 0.017), and an increased Rotterdam CT score in the brain CT (*p* < 0.001). No significant differences in gender, age distribution, types of road user, and initial hypotension were detected between the two groups.

In multivariate analyses of the potential predictors of in-hospital mortality among RTA-related severe TBI, gender, hypothermia, the mGCS score, a prolonged initial prothrombin time, initial hyperglycemia, and the Rotterdam CT score were evaluated. There were the most missing values for hyperglycemia (32.8%), followed by coagulopathy (17.2%) and the Rotterdam CT score (11.7%) (Table 3). Children with a lower initial mGCS score had a higher mortality rate. Each one-point decrease in the mGCS score was associated with a two-fold increase in the odds of mortality (adjusted OR = 2.0, 95% CI: 1.3–3.1, *p* = 0.002). The Rotterdam CT score was also an independent predictor of in-hospital mortality, with a one-point increase in the Rotterdam CT score being associated with a 2.58 increase in the odds of mortality (adjusted OR = 2.58, 95% CI: 1.3–5.5, *p* = 0.006).

## 4. Discussion

TBI is a leading cause of morbidity and mortality in children. RTAs are the most common mechanism of pediatric TBI [3,15]. The injury severity of TBI caused by traffic accidents is more likely to be higher and is associated with higher mortality rates [19]. Our study reveals some clinical characteristics for RTA-related TBI children with higher in-hospital mortality, including presenting to the ED with hypothermia, a lower mGCS score, laboratory findings of an elevated initial prothrombin time and hyperglycemia, and an initial brain CT with a higher Rotterdam CT score. Moreover, lower mGCS and higher Rotterdam CT scores were independent predictors of mortality in these RTA-related severe TBI children who had no severe chest or abdominal injuries.

Motorcyclists were the most common type of road traffic user in this study. Motorcycles are a commonly used type of transportation and were estimated to have a density of about 378 motorcycles/km^2^ in 2017 in Taiwan [34]. In 1997, the motorcycle helmet law was introduced and has effectively decreased the mortality and morbidity from a motorcycle-related head injury [35]. Non-standard helmet use, phone use, and drunk riding were reported risk factors for head injury in motorcyclists [36]. Legislation requiring the use of helmets decreases RTA-related head injuries and fatalities among motorcyclists and bicyclists in young populations [37]. Further safety legislations are important to decrease the incidence of traffic accident-related TBI globally.

Hypothermia observed on arrival at the ED has been associated with poor outcomes in children with severe TBI [14,38]. Hypothermia may result directly from traumatic events, or from the human body’s systemic response to injury. The cause may not only be related to hemorrhagic shock, as subsequent peripheral vasoconstriction and tissue hypoperfusion, or autonomic core temperature regulation dysfunction following extensive brain damage, may also occur in isolated severe cases of TBI [39,40].

Hypotension occurring early after TBI leads to reduced cerebral blood flow and secondary brain injury and was associated with increased mortality in isolated severe TBI children lacking other co-existing injuries to their major organs [41]. Initial hypotension was not significantly associated with increased mortality in this study. This may relate to the exclusion of subjects with co-existing severe chest or abdomen injuries; this resulted in fewer subjects in the hypotension group, and less power to detect a significant association, although there was higher mortality in the hypotension group (50% vs. 14.9%) [42]. Adequate SBP is very important for maintaining cerebral blood because its regulation is even more impaired in younger children [43].

Use of the mGCS score was adopted in this study because of a concern about the difficulty in assessing the full GCS in children with TBI, given the possibility of poor verbal communication. The mGCS score has been proved to have the same predictive ability of mortality as the total GCS score in pediatric TBI [22,44]. Our study further suggests that the mGCS score is an independent predictor of hospital mortality in traffic accident-related severe TBI children. For every one-point decrease in the mGCS score, the odds of mortality increased two-fold.

Hyperglycemia commonly occurs in moderate and severe TBI children, especially in younger ones [24,45]. Our study suggests an association between initial hyperglycemia and increased mortality in severe TBI children, which is compatible with previous studies [25,46]. Elkon et al. reported that persistent hyperglycemia during the first 12 h after injury was an independent risk factor for poor outcomes in TBI children [47]. However, a meta-analysis suggested that glycemic control does not improve mortality but increases the incidence of hypoglycemia in patients with TBI [45]. Initial blood sugar monitoring can alert clinicians about the possibility of poor outcomes in these severe TBI children.

An initial prolonged prothrombin time was also associated with increased mortality in these RTA-related severe TBI children, and such findings have also been reported in previous studies [20,46]. It is known that after severe trauma, hypothermia, acidosis, and hemodilution may occur, which consume coagulation factors and may result in coagulopathy [21]. As with hyperglycemia, although initial coagulopathy has been considered as a risk factor for mortality in pediatric TBI, there is no evidence that correction of trauma-induced coagulopathy could improve the outcome [20].

The severity of the intracranial insult is still the main risk factor in predicting the final prognosis of RTA-related pediatric TBI. Non-contrast CT is the most commonly used brain neuroimaging technique for its rapid acquisition and its ready availability in most EDs. Individual initial brain CT components of subdural hemorrhage, subarachnoid hemorrhage, parenchymal hemorrhage, brain edema, and mass effects had been reported for their association with mortality in pediatric TBI [16], and epidural hematoma was associated with better outcomes [48]. The Rotterdam CT score, established in 2005 [32], is composed of five main CT indices, including the basilar cistern status, presence/degree of midline shift, presence of subarachnoid hemorrhage/intraventricular hemorrhage, and presence of epidural hemorrhage. Kate Liesemer et al. validated the Rotterdam CT score in predicting mortality in pediatric TBI [49]. Our study further suggests that the Rotterdam CT score was an independent predictor of mortality in these RTA-related severe TBI children, with a one-point increment in the Rotterdam CT score being associated with a 2.58-fold increase in the odds of mortality. Recently published guidelines for managing severe pediatric TBI also suggested using the Rotterdam CT score to evaluate brain CT, as a higher score was associated with higher mortality [50].

### Limitation

This study has some limitations. First, the sample size was limited due to the data being obtained from a hospital-based registry, which limits the generalizability of the sample population. Second, due to the nature of the study design, selection bias and missing data could not be completely prevented. We used multiple imputations for the missing data, as valid multiple imputations can reduce bias, even when the proportion of missing data is large [51]. Third, the study period was long, and medical therapeutic options, health policy, and public transportation habits may vary, influencing mortality. Fourth, not all potential predictors of mortality were evaluated. Our study only assessed data in the ED that were routinely documented, well recorded, and clinically reliable. The magnitude of the traffic accidents, the adequate use of protection devices, or not, during transportation, intoxication status (alcohol and drug), and pupil size and reactivity were not evaluated. Although there were several limitations, we offered evidence of associated and independent risk factors for in-hospital mortality in RTA-induced severe pediatric TBI. Further prospective cohort and long-term follow-up studies are warranted in these children.

## 5. Conclusions

The mortality rate of children with RTA-related severe TBI who lacked co-existing severe chest and abdominal injuries was still high. Initial presentation with hypothermia, a lower mGCS score, a prolonged prothrombin time, hyperglycemia, and a higher Rotterdam CT score was associated with mortality. Clinicians should be alert to the higher risk of in-hospital mortality in these children on admission. The mGCS and the Rotterdam CT scores are two independent predictors of in-hospital mortality. Protection of the pediatric head and preventing direct or indirect insults to the brain are important policies in the prevention of RTA-related pediatric TBI and the resulting mortality.

## Figures and Tables

**Table 1:** Demographic and clinical characteristics of the total, mild–moderate, and severe road traffic accident-related traumatic brain injury cases.

Clinical Characteristics	Total	Mild–Moderate TBI	Severe TBI (*n* = 128)	*p*-Value
	(*n* = 618)	(*n* = 490)		
**Gender, *n* (%)**				
Boys	427 (69.09)	311 (67.55)	96 (75)	0.718
Girls	191 (30.91)	159 (32.45)	32 (25)	
**Age (years)**				
Median (25, 75%)	16 (11, 18)	16 (10, 18)	17 (13.5, 18)	**0.004**
**Types of Road User, *n* (%)**				
Pedestrian	80 (13.96)	63 (13.6)	17 (15.6)	0.418
Four-wheeled vehicles	47 (8.20)	42 (9.05)	5 (4.59)	
Motorcycles	397 (69.28)	318 (68.53)	79 (72.48)	
Bicycles	49 (8.55)	41 (8.84)	8 (7.34)	
**Mortality, *n* (%)**				
Yes	23 (3.72)	3 (0.61)	20 (15.62)	**<0.001**
No	595 (96.28)	487 (99.39)	108(84.38)	

**Table 2:** Univariate analyses of association of mortality in children with road traffic accident-related traumatic brain injury.

	Alive (*n* = 108) *n* (%)	Died (*n* = 20) *n* (%)	Test Statistic	*p*-Value
**Gender**				
Boys	84 (87.50)	12 (12.50)	2.84	0.092
Girls	24 (75.00)	8 (25.00)		
**Age (years)**				
Median (25, 75%)	17 (13, 18)	16.5 (14, 18)		0.908
**Type of road user**				
Pedestrian	14 (82.35)	3 (17.65)	1.63	0.694
Four-wheeled vehicles	4 (80.00)	1 (20.00)		
Motorcycles	67 (84.81)	12 (15.19)		
Bicycles	8 (100.00)	0 (0.00)		
**Clinical Presentations:**				
Hypothermia				
Present	3 (42.86)	4 (57.14)	9.68	**0.011**
Not present	105 (86.78)	16 (13.22)		
**Hypotension**				
Present	2 (50.00)	2 (50.00)	3.55	0.120
Not present	103 (85.12)	18 (14.88)		
**Motor component of GCS**				
M5	56 (96.55)	2 (3.45)	41.08	**<0.001**
M4	36 (90.0)	4 (10.00)		
M3	4 (100)	0 (0)		
M2	2 (28.57)	5 (71.43)		
M1	9 (50.00)	9 (50.00)		
**Prothrombin time**				
>1.2	1 (25.00)	3 (75.00)	10.73	**0.013**
≤1.2	88 (86.27)	14 (13.73)		
**Glucose**				
>200	16 (69.57)	7 (30.43)	5.74	**0.017**
≤200	57 (90.48)	6 (9.52)		
**Rotterdam CT score**				
1	6 (100)	0 (0.00)	25.52	**<0.001**
2	20 (95.24)	1 (4.76)		
3	29 (93.55)	2 (6.45)		
4	17 (100)	0 (0.00)		
5	19 (70.37)	8 (29.63)		
6	5 (45.45)	6 (54.55)		

GCS: Glasgow Coma Scale; CT: computed tomography.

**Table 3:** Multivariate predictive models for mortality in severe road traffic accident-related traumatic brain injury children.

	Adjusted OR	95% CI	Z Score	*p*-Value
Gender	0.82	0.18–3.74	−0.25	0.800
Hypothermia	4.26	0.43–41.94	1.24	0.214
Motor component of GCS	2.00	1.28–3.14	3.04	0.002
Prothrombin time	11.32	0.38–336.94	1.14	0.160
Hyperglycemia	4.27	0.66–27.56	1.54	0.126
Rotterdam CT score	2.58	1.31–5.06	2.75	0.006

GCS = Glasgow Coma Scale Score; CT = computed tomography; OR = odds ratio; CI = confidence interval.

## Data Availability

The data presented in this study are available on request from the corresponding author. The data are not publicly available due to the restriction of local law and government policy.
